# Performance Evaluation of Bluetooth Low Energy: A Systematic Review

**DOI:** 10.3390/s17122898

**Published:** 2017-12-13

**Authors:** Jacopo Tosi, Fabrizio Taffoni, Marco Santacatterina, Roberto Sannino, Domenico Formica

**Affiliations:** 1 NeXT: Neurophysiology and Neuroengineering of Human-Technology Interaction Research Unit, School of Medicine, Università Campus Bio-Medico di Roma, 00128 Rome, Italy; f.taffoni@unicampus.it (F.T.); d.formica@unicampus.it (D.F.); 2 Unit of Biomedical Robotics and Biomicrosystems, School of Engineering, Università Campus Bio-Medico di Roma, 00128 Rome, Italy; 3 STMicroelectronics, 20864 Agrate Brianza (MB), Italy; marco.santacatterina@st.com (M.S.); roberto.sannino@st.com (R.S.)

**Keywords:** Bluetooth Low Energy (BLE), performance evaluation, wireless sensor network, wearable technology, Internet of Things (IoT)

## Abstract

Small, compact and embedded sensors are a pervasive technology in everyday life for a wide number of applications (e.g., wearable devices, domotics, e-health systems, etc.). In this context, wireless transmission plays a key role, and among available solutions, Bluetooth Low Energy (BLE) is gaining more and more popularity. BLE merges together good performance, low-energy consumption and widespread diffusion. The aim of this work is to review the main methodologies adopted to investigate BLE performance. The first part of this review is an in-depth description of the protocol, highlighting the main characteristics and implementation details. The second part reviews the state of the art on BLE characteristics and performance. In particular, we analyze throughput, maximum number of connectable sensors, power consumption, latency and maximum reachable range, with the aim to identify what are the current limits of BLE technology. The main results can be resumed as follows: throughput may theoretically reach the limit of ~230 kbps, but actual applications analyzed in this review show throughputs limited to ~100 kbps; the maximum reachable range is strictly dependent on the radio power, and it goes up to a few tens of meters; the maximum number of nodes in the network depends on connection parameters, on the network architecture and specific device characteristics, but it is usually lower than 10; power consumption and latency are largely modeled and analyzed and are strictly dependent on a huge number of parameters. Most of these characteristics are based on analytical models, but there is a need for rigorous experimental evaluations to understand the actual limits.

## 1. Introduction

Bluetooth Low Energy (BLE, Bluetooth 4, Bluetooth Smart) is an innovative technology, developed by the Bluetooth Special Interest Group (SIG), which aims to become the best alternative to the huge number of standard wireless technologies already existing and widespread on the market (i.e., IEEE 802.11b (Wi-Fi), ZigBee, ANT+ and Bluetooth Classic (Bluetooth 3.0, Basic Rate/Enhanced Data Rate)).

The synergy between good performance and ubiquitous diffusion (today, BLE is available in all PCs, tablets and smartphones) makes BLE an excellent candidate for a great variety of applications: in the medical field for e-health applications [1,2,3], e.g., in a body area network [4] (using ECG [5,6], a heart rate sensor [7,8], a blood flowmeter [9], EMG for prosthetic hand control [10] and an IMU sensor used for early diagnosis of Parkinson’s disease [11]; it is also used to monitor respiration, activities and falls [8]), in automotive applications [12,13], in voice communications [14], for kinematic tracking [15,16], in domotics for healthcare environments and smart houses [17,18,19,20], for transmission of M-IMU data in game controlling [21], in security systems [22,23], to understand crowd dynamic [24], and so on. It has been successfully used for position detection and distance measurement using beacon communication. In this modality, different devices or sensors, provided with the BLE interface and placed in a structured environment, are programmed to send broadcast messages (described in Section 2.2.1) so that listener devices (e.g., user’s mobile device) can receive them. In this way, it is possible to send some pieces of information about the surrounding area [25], or to detect the user position [26,27,28,29,30,31,32], or to measure the distance between sensor devices in the environment [33,34], or also to detect the presence of devices, and so people [22,24,35,36,37,38]. Moreover, thanks to its versatility and low power consumption, BLE has also been applied in Internet of Things (IoT) technologies [39], for example transmitting Internet Protocol Version 6 (IPv6) packets over Low power Wireless Personal Area Networks [40,41,42] (6LowPAN) in health monitoring application [43]. This has also been possible thanks to the 6LowPAN work group of the Internet Engineering Task Force (IETF) that has proposed several standards and drafts to specify the header compression scheme for IPv6 packet delivery in low power wireless networks [44]. This fact has also led to the definition of specific function packs, implemented in dedicated MCUs, which allow one to connect 6LowPan IoT nodes via BLE interfaces [45].

The studied examples strongly benefit from BLE characteristics, in particular its low power consumption, which allows it to be embedded in small devices with low-charge and small batteries, which can also last a few years. For all these reasons, BLE is a good candidate to be a revolutionary technology in the present market of wireless communications.

Notwithstanding such promising applications, the BLE still lacks a complete and systematic analysis of its real performance under different experimental conditions, which could help designers to develop optimized devices for specific applications. This review’s purpose is to obtain a useful compendium of BLE, describing its main specifications and characteristics and proposing a roadmap to a systematic characterization of its performance, in order to pave the way for further studies.

In addition to this, in late 2016, Bluetooth SIG released Bluetooth 5 specifications. This new Bluetooth technology offers improvements in performance, such as range, data rate and advertising channel functionality, and some studies have already tried to find out its characteristics [46,47]. Our work can also help further studies to systematically analyze this new technology, as already done with BLE.

This work is organized as follows:

- Firstly, in Section 2, we describe the main frames and functions of the BLE protocol stack, analyzing in detail how the communication works, how a packet is structured and how the possible network typologies are.
- Then, in Section 3, we systematically review the works available in the literature about the use of BLE, providing a common theoretical framework to discuss in detail the main remarks observed in these studies and defining guidelines for the BLE setting in different conditions of use.
- Finally, in Section 4, we summarize studies on the main characteristics, uses and limits of BLE, trying to define guidelines on what is already consolidated in the literature, what are the open issues and suggesting what could be the next utile investigations on this technology.

## 2. BLE Functioning

### 2.1. BLE Protocol Stack

**Figure 1:**
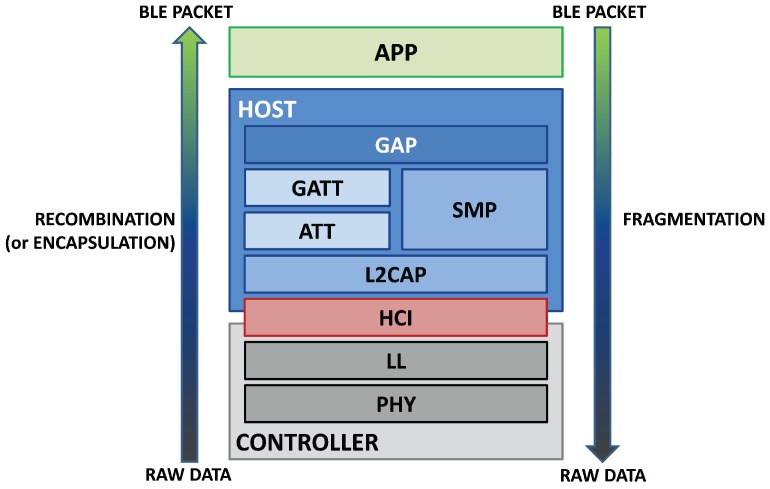
BLE protocol stack. The three main blocks are the *Controller* (grey), the *Host* (blue) and the *App* (green). The *HCI* (red) is the interface that manages the communication between the *Controller* and the *Host*. The rectangular frames represent the different layers of the protocol, and they are ordered in a stack, which starts from the bottom, with the PHY part, and ends at the higher level, that is the *App*. The arrows show how encapsulation and fragmentation work. Adapted from [48].

The BLE protocol is structured in a stack composed of three main blocks [48,49,50,51], as shown in Figure 1:

- The *Application* (*App*) is the highest block of the stack, and it represents the direct interface with the user. It defines some profiles thanks to which different applications, which reuse common functionality, are able to interoperate. These application profiles are specified by the Bluetooth SIG and encourage interoperability between devices from different manufacturers. Bluetooth specification allows also defining vendor-specific profiles for use cases not covered by the SIG-defined profiles.
- The *Host* includes the following layers:
  - Generic Access Profile (GAP)
  - Generic Attribute Profile (GATT)
  - Logical Link Control and Adaptation Protocol (L2CAP)
  - Attribute Protocol (ATT)
  - Security Manager Protocol (SMP)
  - Host Controller Interface (HCI), *Host* side
- The *Controller* is structured in the following layers:
  - Host Controller Interface (HCI), *Controller* side
  - Link Layer (LL)
  - Physical Layer (PHY)

**Figure 2:**
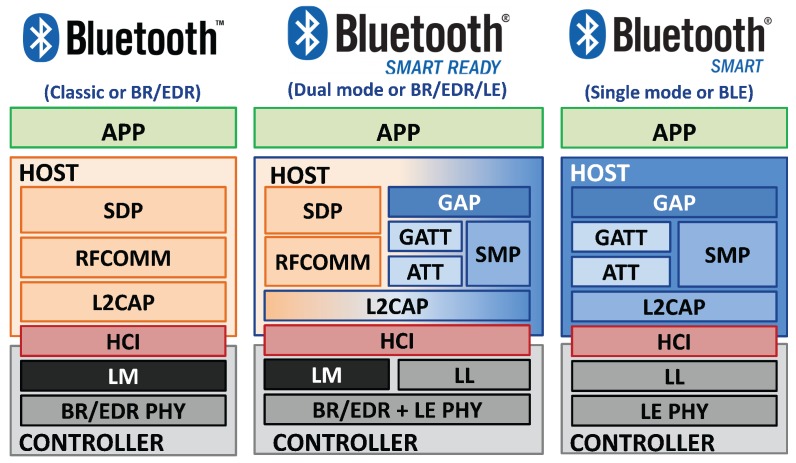
Configuration between Bluetooth versions and device types. On the left is the protocol structure of the BR/EDR, while on the right is the BLE. In the middle is the protocol stack of a device compatible with both Bluetooth versions; this type is called *Smart Ready* or *Dual Mode*. Adapted from [52].

Each layer in the protocol incorporates its lower layer. The raw data, acquired from the antenna, are consequently encapsulated in a standard BLE packet, shown by the arrow on the left. On the other side, a BLE packet that shall be sent by a transmitter is fragmented in raw data and then managed by the PHY layer, as the arrow on the right shows. The BLE architecture has maintained some common parts of Classic Bluetooth, in order to allow the development of devices compatible with both standards (*Smart Ready* devices). In Figure 2 are shown the protocol architectures of Classic Bluetooth, BLE and *Smart Ready* devices, pointing out different and equal blocks between the protocols. As can be seen in the figure, the dual mode shares some common parts with both standards (PHY, HCI, L2CAP and *App* layer), while other layers are typical of the Basic Rate/Enhanced Data Rate (BR/EDR) (Link Manager Protocol (LM), Radio Frequency Communication (RFCOMM) and Service Discovery Protocol (SDP)) or belong to the BLE only (LL, ATT, GATT, SMP and GAPP).

In the following sections, all the layers of the protocol stack will be described in detail.

#### 2.1.1. Physical Layer

**Figure 3:**
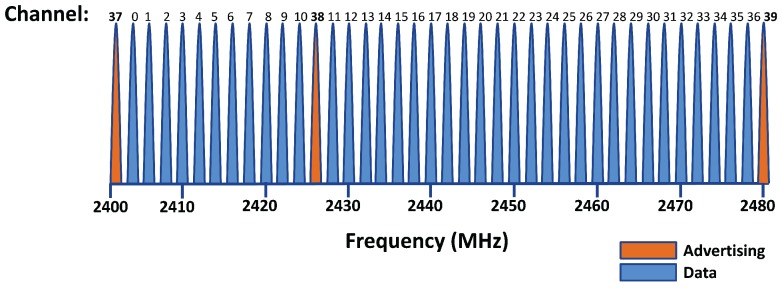
BLE frequency channels. It can be noticed that channels from 0–36 are assigned to data transmission in *connections* (blue), while the three remaining channels, from 37–39, are used as advertising channels, shown in orange. How channels are positioned in the frequency band is shown in the *x*-axis: the first channel, 37, is centered at frequency 2402 MHz, while the last one, the 39th, is centered at 2480 MHz. Adapted from [48].

The BLE technology is designed to operate in the Industrial, Scientific and Medical (ISM) band included in 2.4–2.5 GHz, the same as BR/EDR and Wi-Fi. In particular, the BLE radio band goes from 2.4000 GHz–2.4835 GHz, and it is divided into 40 channels, as can be seen in Figure 3. These channels have center frequencies 2402 + k × 2 MHz, where k = 0, …, 39. Three of these channels (37, 38 and 39) are reserved for advertising packets (Section 2.2.1), while the other 37 are used to exchange data packets in connections (Section 2.2.2).

To avoid interference and fading with other wireless communications in the same radio band, BLE implements an Adaptive Frequency Hopping [53,54], which is a strategy that defines in a pseudo-random way the communication channel used by the two main characteristics of the link.

It is mandatory that PHY work at 1 Mbps (LE 1M PHY) [53]; in this case, each bit transmitted corresponds to a single symbol (uncoded transmission). Using an error correction coding, more bits can be associated with only one symbol, which implies a bit rate of 500 kbps and 125 kbps, when the coding scheme uses respectively two or eight symbols per bit. An optional radio data rate that PHY supports is 2 Mbps (LE 2M PHY), but in this case, it works with uncoded data only.

All these parameters lay the foundation for the throughput evaluation, described in Section 3.1.

BLE PHY also defines the limits for the radio transmit power, which are between the minimum of 0.01 mW (−20 dBm) and the maximum of 10 mW (+10 dBm) [53,55]. In order to optimize the power consumption, along with reducing the interferences and increasing the range of the signal, it is possible to locally change the output power control of the device. The transmit power is the main feature useful to model the maximum range of transmission of BLE (Section 3.5).

#### 2.1.2. Link Layer

The LL is the part of the stack that directly interfaces with the PHY; indeed, it is composed of a combination of a hardware (HW) and a software (SW) part. The LL defines the type of communications that can be created between BLE devices through the managing of the link state of the radio. LL also defines the different roles a device can play, i.e., *master*, *slave*, *advertiser* and *scanner* (described in Section 2.2).

Usually, the LL is implemented in HW by silicon vendors to avoid overloading the Central Process Unit (CPU) responsible for managing all the SW layers of the stack. Its functionalities are easily automated, but computationally expensive, and they usually are:

- Preamble, Access Address and air protocol framing.
- Cyclic Redundancy Check (CRC) generation and verification.
- Data whitening.
- Random number generation.
- Advanced Encryption Standard (AES).

#### 2.1.3. Host Controller Interface

The HCI is a standard protocol that takes care of the communication between the *Controller*, that is the lowest part of the protocol, and the *Host*, i.e., the core of the BLE protocol stack, which manages the communication between the HW and the user application. Therefore, its role is to define a set of commands and events in order to translate raw data into data packets to send them via serial port to the *Host* layer, and vice versa. That has been necessary because the protocol performs a modularity, and for this reason, it does not incorporate *Controller*, *Host* and *Application* in a single package.

#### 2.1.4. Logical Link Control and Adaptation Protocol

The L2CAP is a protocol in common with BR/EDR which acts as a multiplexer; it handles the data from lower layers (LL for BLE and LM for BR/EDR) and encapsulates them into the standard BLE packet format, according to the upper layers (ATT and SMP for BLE and RFCOMM for BR/EDR), and vice versa; these processes are respectively called *recombination* (or *encapsulation*) and *fragmentation*, as shown in Figure 1.

#### 2.1.5. Security Manager Protocol

The SMP is composed of several security algorithms in order to encrypt and decrypt data packets. It defines two main roles during the establishment of a connection: the *initiator* and the *responder*, which will correspond respectively to the *master* and the *slave* (Section 2.2), once the connection is established. Further details of the SMP procedures, such as pairing, bonding and encryption re-establishment, can be found in [48].

#### 2.1.6. Attribute Protocol

The ATT defines the roles of a *client-server* architecture [56], where the *client* is the one that requests data from the *server*, which, in turn, sends data to *clients*. Usually, these roles correspond respectively to the *master* and the *slave* defined in the LL, Section 2.1.2, but in general, a device could be a *client*, a *server*, or both, irrespective of whether it is a *master* or a *slave*. The ATT also performs data organization into attributes, to which is assigned a handle, a Universally Unique Identifier (UUID), a set of permissions and a value. This protocol is encapsulated in the GATT, Section 2.1.7, which uses the roles defined in the ATT to perform connections.

#### 2.1.7. Generic Attribute Profile

The GATT encapsulates the ATT layer, and its main role is to establish how to exchange all profiles’ information and data in a BLE link. Profiles are definitions of possible applications and specify general behaviors that Bluetooth devices use to communicate with other Bluetooth devices. Profiles are built on the Bluetooth standard to more clearly define what kind of data a Bluetooth module is transmitting. These data are organized in a hierarchical structure composed of sections called *services*, which, in turn, group data into containers called *characteristics*.

GATT defines two roles in a connection, *client* and *server*, which correspond to those described in the ATT protocol (Section 2.1.6). The Bluetooth SIG defines some standard *services* and *characteristics* represented by a 16-bit address format, but the strength of BLE is that it lets the manufacturers define their own *services* using a 128-bit UUID in order to adapt this technology to brand new and original applications.

**Figure 4:**
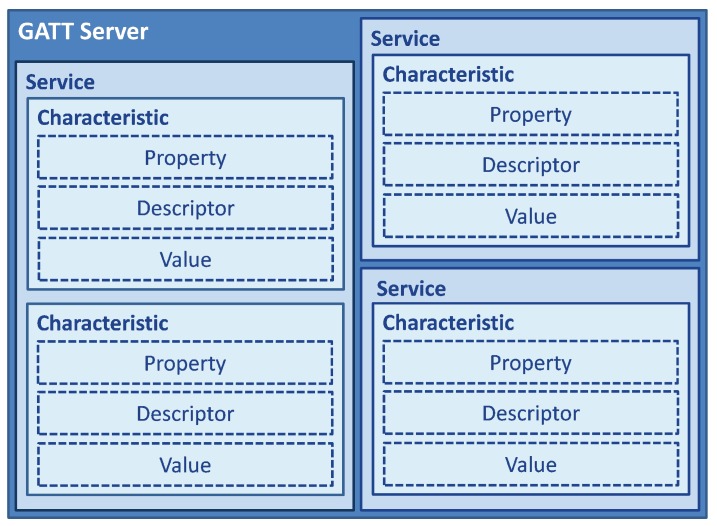
GATT data hierarchy. Immediately before the connection, the GATT *server* exposes its *services* and *characteristics*. As shown in this figure, *services* and *characteristics* are defined in order to form a logical data structure. Moreover, each *characteristic* exposes its properties, a descriptor that defines what it does, and the data value. Adapted from [52].

During the connection establishment, the *server* exposes its *services* and *characteristics* to the *client* in order to define how the connection will be structured. The logical structure of the GATT server profile, which includes several *services* and *characteristics*, is shown in Figure 4.

A *service* is basically a container that conceptually groups related attributes, while *characteristics* are the attributes included in a *service*, and each of them is used to communicate a specific type of data. This service-oriented paradigm is a further abstraction on top of the *client-server* architecture where the *services* have a defined behavior that will always give the same type of response [56].

*Characteristics* contain the data value, a descriptor, which gives additional information about the *characteristic*, its value and some properties. These properties indicate to the *client* which operations are allowed to be performed on the *characteristic*; the properties used most are:

- *Broadcast*: this allows sending data to BLE devices using advertising packets, as described in Section 2.2.1.
- *Readable*: if set, the client can only read the *characteristic* value.
- *Writable*: with this property, the client can only write a new value on the *characteristic*.
- *Notifiable*: when it is set, the *client* receives a notification if the *server* updates the *characteristic*, so that it can read the new value.

**Figure 5:**
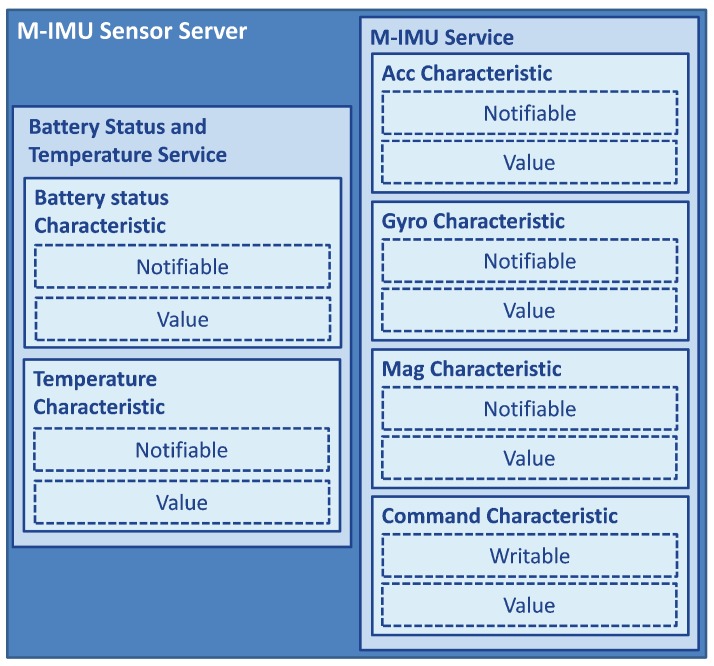
GATT data hierarchy, relative to the example described in Section 2.1.7.

An example, useful to clarify the structure of the GATT *server*’s profile hierarchy, could be the one described here following. Let us assume that we need to send data from a Magneto-Inertial Measurement Unit (M-IMU) (i.e., a unit that collects three different sensors: an accelerometer, a gyroscope and a magnetometer), that is the *master*, to a mobile device such as a smartphone or a tablet, which represents the *slave*. Let us also assume that the board has an embedded battery, in order to make the device portable, and a temperature sensor: in fact, it is important to know these data since the M-IMU output is influenced by the variation of temperature, for example due to the battery overheating. In this example, it could be useful to construct the profile with two different *services*, as shown in Figure 5.

- *M-IMU service*: This manages all the data referred to the M-IMU, and it is structured into four different *characteristics*. Three notifiable *characteristics* perform the task to send data from the sensor to the *central* device; one is referred to the accelerometer data, one for the gyroscope and the last one for the magnetometer. The last *characteristic* is writable so that the *central* node can modify some properties of the M-IMU, for example the sampling frequency or how many of the three sensors are transmitting.
- *Battery Status and Temperature service*: This has two notifiable *characteristics*, used to send data relative to the remaining battery charge and the temperature. In order to preserve energy, it could send data with a rate lower than the one of the *service* previously described. This is another reason why it is better to correctly manage the GATT logical structure; in this way, it is possible to separate data transmission depending on the specific use.

Another notable example of the utility of the BLE data structure is provided by Gentili et al. [14], where the *service* and the two *characteristics* are described in detail. In this case, the *service*’s main functionality is to send audio between two nodes; both *characteristics* are notifiable; indeed, their application consists of a simplex mode transmission where the *slave* can only send audio data to the *master*. Both *characteristics* are encapsulated in the same *service* because they join the same application, but while the first one sends the audio data compressed, the second one transmits the information needed to let the *master* decompress them.

#### 2.1.8. Generic Access Profile

In the BLE stack, the GAP is collocated at the highest level of its core; it specifies device roles, modes and procedures, in addition to managing the connection establishment and the security. It interfaces directly with the *Application* layer and thus to the user, which can define all the parameters that the network needs. Moreover, it provides the link between the user and all of the stack protocol; indeed, it implements and controls all the lower protocols. All the roles and the procedures defined in the GAP are described in Section 2.2.

### 2.2. BLE Communication

Investigating the structure and the functioning of a BLE network is important to understand how it communicates and the roles a device can play. BLE devices can communicate using two main modalities: *broadcasting* and *connections* [54,57]. The parameters defined in this subsection are very important; as a matter of fact, their settings deeply influence BLE performance, described in Section 3.

#### 2.2.1. Broadcasting

In the connectionless *broadcasting*, advertising packets can be sent out in one way, from a single device to any scanning or receiving device in the listening range. *Broadcasting* is the fastest way to transmit data to more than one peer at the same time, but its major limitation is that it is not suitable for sensitive data because it has no security or privacy controls.

*Broadcasting* packets have two main purposes: the first one is to send advertising packets to applications which do not need a full active connection; the second one is when a *master* sends connectable advertising packets to discover *slaves* available for connection.

*Broadcasting* defines two roles, specified in GAP [58]:

- *Broadcaster (Advertiser)* periodically sends advertising packets to any device able to receive them.
- *Observer (Scanner)* continuously scans, at periodic intervals, if there are available advertising packets to receive from a *broadcaster*.

A property that must be set for the *broadcaster* is the *Advertising Interval (advInterval)*: it represents the rate at which the advertising packets are sent. Otherwise, on the *observer*’s side, the *Scan Interval (scanInterval)* must be set, i.e., the rate at which the *scanner*’s radio turns on, and the *Scan Window*, that is the time the radio keeps on scanning per each *scanInterval*. These two parameters set the amount of time the radio must be turned on; therefore, they have a deep impact on the current consumption, so they must be set carefully.

**Figure 6:**
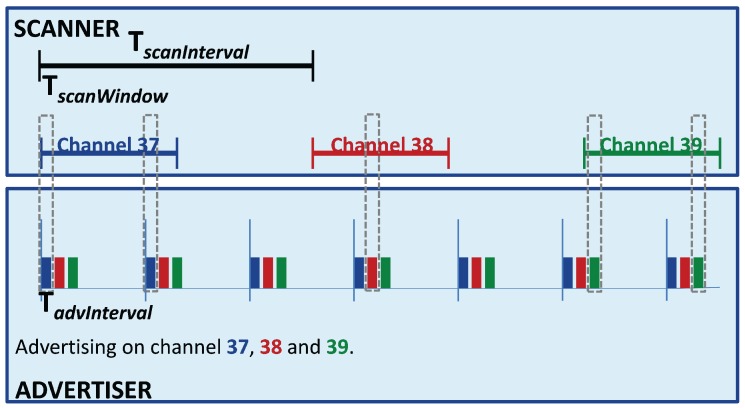
This figure shows how the advertising and scanning mechanisms work. In the upper part is shown the *scanner*; it scans on the three advertising channels (37, 38, 39, colored respectively in blue, red and green), switching the scanning channel within a period called T${}_{scanInterval}$. The effective scanning period, per each channel, lasts for a time called T${}_{scanWindow}$. On the other hand, the *advertiser*, shown in the bottom part of the Figure, sends a burst of three advertising packets, one for each advertising channel, with a specific period (T${}_{advInterval}$).

As defined in Section 2.1.1, BLE specifies three channels reserved for the *broadcasting*, which means that the *broadcaster* sends the same packet in all three channels for each *advInterval*, as shown in Figure 6, while the *scanner* changes the channel where to scan in each *scanInterval*.

#### 2.2.2. Connections

A *connection* is a permanent, periodical data exchange of packets between two devices [52]. The *connection* is private, and it could also be protected with security provisions. In *connections*, there are two different roles involved, defined in the GAP [57]:

- The *Central (master)* scans for connectable advertising packets and initiates the *connection*. When the *connection* is active, the *central* manages all the setting and starts a periodical packet exchanges.
- The *Peripheral (slave)* periodically sends connectable advertising packets and accepts *connections* initiated by the *master*. When the *connection* is established, it follows the settings exposed by the *central* and exchanges data with it.

As already said in Section 2.2.1, the beginning of a *connection* works as a *broadcasting* mode. In this case, the *broadcaster*, which sends connectable advertising packets, is also called the *responder* and will become the *slave*, while the *observer* is also called the *initiator* and will become the *master*, as described in Section 2.1.5.

**Figure 7:**
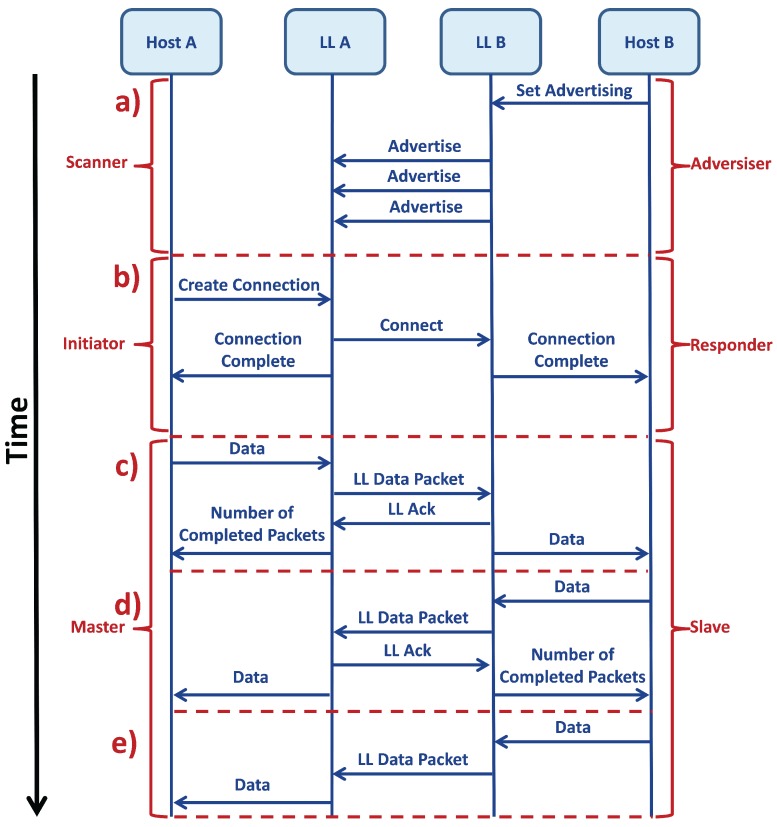
This figure shows how two devices (A and B) communicate through BLE. The two vertical blue lines on the left represent the *Host* layer and the LL of the device A, while the two lines on the right represent the same two layers relative to the device B. Blue arrows represent the communication messages between layers and devices. The black arrow on the left shows that the time increments scrolling the figure to the bottom. Red braces indicate the specific role of the devices A and B during the different parts of the communication. Part (**a**) of the figure shows the *advertising* mechanism, in which the *advertiser* (B) sends a message from the *Host* to the LL in order to enable the sending of advertising packets. The *scanner* (A) receives these packets and prepares itself for the *connection*. In (**b**) is shown the *connection* establishment where the *initiator* (A) sends a message in order to create the *connection*, firstly from the *Host* to its own LL and then to the other device, the *responder* (B). At the end, if the *connection* is established correctly, the two LLs send a message to the respective *Host* layers in order to confirm the correct creation of the *connection*. Starting from (**c**), the *connection* is created, and the two devices are called *master* (A) and *slave* (B). In (**c**), the *master* sends data packets to the *slave*, writing on a writable *characteristic*. In this case, data packets are sent from the *master Host* layer to its own LL and then to the *slave*. During this process, an Acknowledgment packet (ACK) is sent back to the *master* in order to communicate to the *Host* layer if and how many packets were correctly transmitted. This type of communication, with the ACK packet, is called *round-trip*. A similar process is shown in (**d**), where the *slave* writes on its readable *characteristics* and the *master* reads the data. Also in this type of communication, there is the transmission of an ACK packet, so this is also a *round-trip* communication. In (**e**), a *one-way* communication is represented, where the *slave* communicates with the *master* using a notifiable *characteristic*, which means there is no ACK packet.

The setup of a *connection* [59] is shown in Figure 7. Before the *connection* starts, the *peripheral* is in advertising mode and sends connectable advertising packets, while the *central* unit is in discovery mode, looking for some connectable packet from any reachable *slave*. As soon as the *master* finds the *slave*, it sends a *connection* request, and after the *peripheral* accepts the request, the *connection* is created. At this moment, the two modules can communicate, sending data packages between them; it is important to notice that, although the *connection* parameters are set only by the *master*, data can be sent by both roles during each *connEvent*; it only depends on the properties of the *characteristics* (Section 2.1.7).

**Figure 8:**
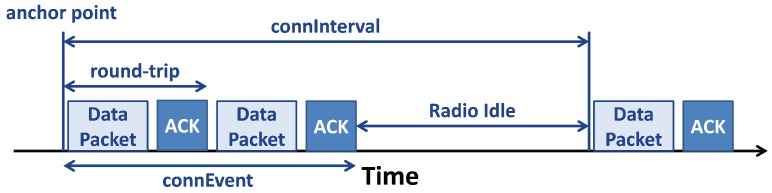
Example of a *round-trip* data communication in a *connection* with the transmission of data packets and ACKs. In this figure are described all the *connection* parameters.

A *connection* between a *master* and a *slave*, shown in Figure 8, follows predefined times: the time in which the *master* exchanges data packets with the *slave* is called *Connection Event (connEvent)*, while the rest of the time, when the communication is off, is the *Radio Idle*. The start of a *connEvent* is called the *anchor point*; at the *anchor point*, the *master* shall start to transmit packets to the *slave*.

Other parameters, useful to describe the BLE *connection*, can also be used to set up the *connection* itself [59]:

- *Connection Interval (connInterval)* is the time between the beginning of two consecutive *connEvents*; in other words, it is the sum of *connEvent* and *Radio Idle*. The *connInterval* shall be a multiple of 1.25 ms in the range of 7.5 ms to 4.0 s.
- *Connection Supervision Timeout (connSupervisionTimeout)* is the maximum time that can flow without receiving two valid packets, before the *connection* is lost. The *connSupervisionTimeout* should be a multiple of 10 in the range of 100 ms to 32,000 ms.
- *Connection Slave Latency (connSlaveLatency)* is the amount of *connEvents* that can be skipped without the risk of a disconnection. The value of *connSlaveLatency* should not cause a *connSupervisionTimeout*, and it shall be an integer in the range of zero to ((*connSupervisionTimeout*/(*connInterval* × 2)) − 1). Moreover, *connSlaveLatency* shall not be less than 500, and when it is set to zero, the *slave* device shall listen at every *anchor point*, without loosing the *connection*.

As can be seen in Figure 7 and Figure 8, a data packet exchange is usually followed by another packet called Acknowledgment (ACK), i.e., a packet without data, which provides error recovery capabilities. Depending on the type of communication, i.e., when data are sent through a notifiable *characteristic*, the ACKs are not sent.

There are two main communication modalities the *master* can use to exchange data with the *slave* [50]:

- In *one-way* ATT communication, the *slave* sends a simple notification in response to a poll from the *master*. This is typical of the communication through notifiable *characteristics*.
- In *round-trip* ATT communication, the *master* firstly asks for data to the *slave*, then this one transmits a response. The difference is that both messages, the request and the response, generate an ACK. The interval of time between the beginning of two consecutive data packet, including the ACK, is called $T_{round-trip}$.

### 2.3. BLE Packet

**Figure 9:**
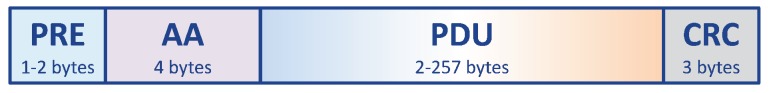
BLE packet structure. The packet has one or two bytes of PRE, depending on the radio data rate, four bytes of AA, from two to 257 bytes of PDU and three bytes of CRC.

As described in Section 2.2, BLE allows two types of communication (i.e., *broadcasting* and *connection*), which implies two different packet typologies, which share a common structure, as shown in Figure 9. This structure is divided into four mandatory subsections, defined in the LL BLE Specification [54,58], and described as follows:

- The *Preamble (PRE)* length depends on the radio data rate, and it is equal to one or two bytes, respectively, if the connection works on LE 1M PHY or on LE 2M PHY, described in Section 2.1.1. It is a very simple sequence of bits used by the receiver to set its automatic gain control and determine the frequency corresponding to the radio data rate itself.
- The *Access Address (AA)* is the group that includes the four following bytes and identifies the communication on a physical link, and it is used to exclude packets directed to different receivers.
- The *Protocol Data Unit (PDU)* range is from two to 257 bytes, and its length is strictly dependent on the type of communication used; it is described more in detail below.
- The *CRC* is a subsection of three bytes, which checks the presence of errors, analyzing the *PDU* only, which could have been generated during packet transmission. A detailed analysis of error correction techniques, with a specific focus on BLE CRC, has been proposed in [60].

**Figure 10:**
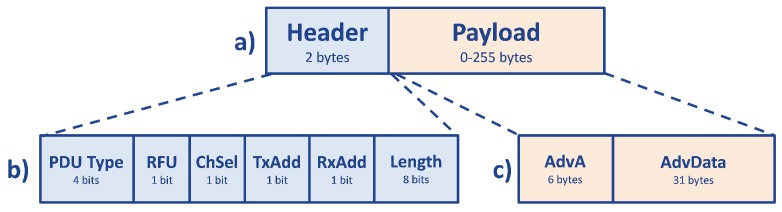
PDU structure of an advertising packet. In (**a**) the first two bytes represent the Header, while the other bytes are the effective payload of the packet. The Header, showed in detail in (**b**), is composed of 4 bits of the PDU Type, 1 bit of RFU, 1 bit of ChSel, 1 bit of TxAdd, 1 bit of RxAdd and 8 bits of length. Then, in the case of advertising packets, only 37 bytes of the remaining 255 of payload are filled. The first six bytes are the AdvA and the last 31 form the AdvData, as shown in (**c**).

The *broadcasting* channel PDU (shown in Figure 10) has a 16-bit header and a variable size payload, from zero to 255 bytes. The header contains four bits that indicate the PDU type, because there are several types of *broadcasting* packets. The following bit is Reserved for Future Use (RFU), while the next three bits are referred to Channel Selection (ChSel), Transmitter Address (TxAdd) and Receiver Address (RxAdd), and they are exploited depending on the PDU type used, described in detail in [58]. Finally, the last eight bits in the header indicate the length of the payload. Except for some specific task, the *broadcasting* data packet is sent by the *advertiser*, and the payload is composed of six bytes of the *Advertiser*’s device Address (AdvA), which contains the *advertiser*’s public or random device address (depending on if TxAdd is equal to zero or one), and a maximum of 31 bytes of Advertising Data (AdvData), i.e., the effective payload. In case this payload is not sufficient for the specific application, the *scanner* can request for another packet of 31 bytes maximum; this procedure is called active scanning [48,54], and if used, it is declared in the PDU type. A detailed description and analysis of passive and active scanning and their performance is proposed in [61].

**Figure 11:**
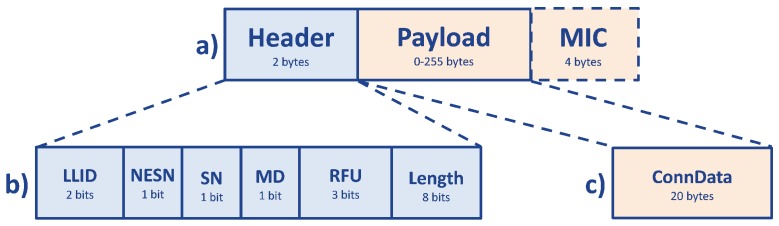
PDU structure of a BLE connection packet. In (**a**) the first two bytes represent the Header, while the other bytes are the effective payload of the packet; in addition to this there are four optional bytes at the end, existing only in LL encrypted connections, which are the MIC. The Header, showed in detail in (**b**), is composed of 2 bits of LLID, 1 bit of NESN, 1 bit of SN, 1 bit of MD, 3 bits of RFU and 8 bits of Length. Then, in the case of BLE connection packets, only 20 bytes of the remaining 255 of payload are filled, and these bytes represent the ConnData, as shown in (**c**).

On the other side, a *connection* packet (Figure 11) is composed of a two-byte header, which contains several parameters, described in detail in [58]: the Logical Link Identifier (LLID) (2 bits), the Next Expected Sequence Number (NESN) (1 bit), the Sequence Number (SN) (1 bit), More Data (MD) (1 bit), the RFU (3 bits) and the the payload Length (8 bits). In particular, the LLID value indicates if the PDU contains data or control messages. The maximum effective payload for data packets (ConnData) is 20 bytes, set by the BLE specification [52]. The limit of 20 bytes is not dependent on the LL, but it is a GATT layer specification, where it is defined that the Maximum Transmission Unit (MTU), for a packet sent between a *master* and a *slave*, shall be lower than 23 bytes; then, excluding the two-byte header, the effective payload may be 20 bytes maximum [62]. Moreover, there is an optional Message Integrity Check (MIC) value of four bytes, which is used to authenticate the data PDU in an LL encrypted connection.

### 2.4. BLE Network Topology

**Figure 12:**
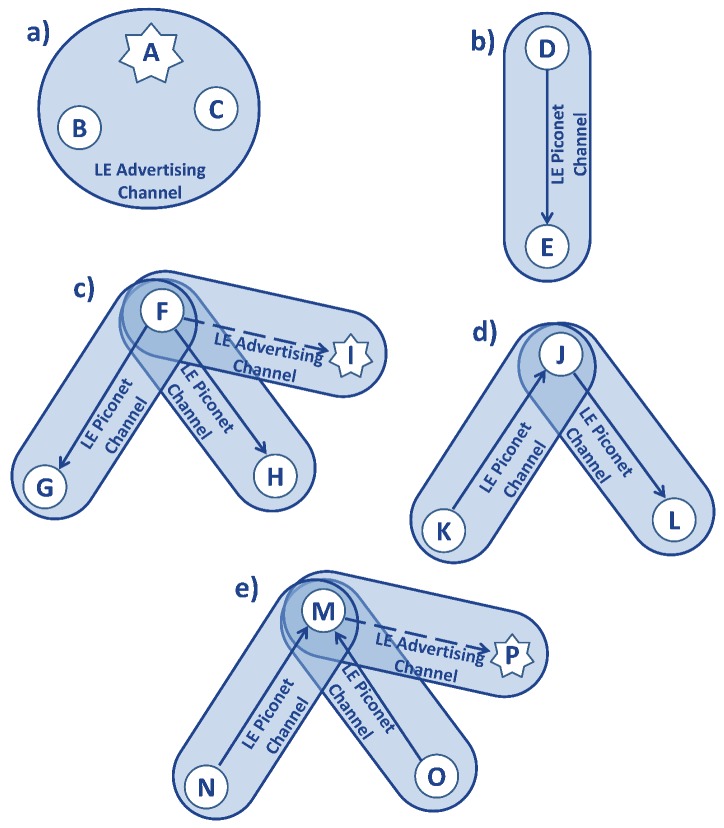
Example of BLE topology [59]. In the figure, solid arrows point from *master* to *slave*; dashed arrows indicate a connection initiation and point from *initiator* to *responder*. Each device is represented with a capital letter; devices that are connected are represented with a circle, while devices that are advertising are indicated using stars. Group (**a**) in Figure 12 is a simple *broadcasting* topology, where A is an *advertiser*, while B and C are *scanners*, which are using a BLE advertising physical channel. Group (**b**) is a basic *piconet*, with only one physical channel, where D acts as *master* and E as *slave*. In group (**c**), the *master* is F, and it is using two *piconet* physical channels with *slaves* G and H. Device F is also the *initiator* of the connection with device I, which is advertising with connectable advertising packets on the advertising physical channel; device F can start the connection and add *slave* I to its *piconet*. A network topology like this one, with only one *master* and several *slaves*, is called a star network. In *scatternet* (**d**), device J is using one LE physical channel with K and one with L. J is the *master* in the *piconet* with L and the *slave* in the one with K. In *scatternet* (**e**), device M is the *slave* of two physical channels, whose *masters* are N and O. Device P is advertising using a connectable advertising event on the advertising physical channel, and the device M is the *initiator*; when the connection is formed, M will result in being the *master* of this link.

A BLE basic network, composed by a *master* and a *slave*, is called *piconet*. With the updating to Version 4.1 of the Specification of the Bluetooth System, each device has the capability to perform simultaneously both roles, *master* and *slave*, in different *piconets*. This type of network is called *scatternet*. In the Bluetooth Specification [58], some types of BLE topology are shown in detail, as summarized in Figure 12.

A more complex network topology, which offers path diversity that copes with radio propagation impairments and node failures, is the *mesh* network. This type of network has been introduced in BLE specifications starting from Version 4.1, where a node has been enabled to act as *master* and *slave* simultaneously, as already said in this section. Some papers in the literature have already studied different possibilities of how to implement a BLE mesh network and also how to address the main correlated problems, such as the dynamic address allocation, network topology mapping and data routing [63]. In detail, Darroudi et Gomez [64] provided a complete taxonomy of BLE mesh network solutions present in the state of the art.

## 3. BLE Performance

In this section, we examine the main performances of BLE and how they have been analyzed in literature. The main characteristics, examined in the following sections, are: throughput, the number of devices that may be connected to a single *master*, power consumption, connection and transmission latency and the maximum reachable range.

In Table 1 we summarize the main parameters introduced in Section 2, in order to better understand the BLE performance analysis made in the following sections.

### 3.1. Throughput

The first factor that influences throughput is the data rate implemented in the radio protocol. The BLE radio data rate specification, defined in Section 2.1.1, is set to 1 Mbps.

Knowing the total time needed to send a single packet ($T_{round-trip}$), that is ~675 μs [65], the maximum theoretical number of *round-trips* ($N_{round-trip}$) per *connInterval* can be calculated as the floor of the ratio of *connInterval* and $T_{round-trip}$:

(1) $$N_{round-trip}=⎣\frac{\mathrm{connInterval}}{T_{round-trip}}⎦$$

Using Equation (1), it is possible to obtain the maximum number of packets for the minimum *connInterval* of 7.5 ms, which is 11.

That means the maximum throughput ($\delta_{Max}$) is equal to the maximum number of bits ($N_{Max}$) per each *connInterval* divided by the *connInterval* itself, and it is equal to:

(2) $$\delta_{Max}=\frac{N_{Max}}{\mathrm{connInterval}}=\frac{N_{round-trip}\times 20bytes\times 8bits}{\mathrm{connInterval}}$$

In the case of errors during the transmission, the throughput (δ) will be reduced by a factor called the Bit Error Rate (BER), as shown in Equation (3). The BER corresponds to zero when there are no errors in the communication; otherwise, when all the packets are lost, it is equal to one. In the BLE protocol, if a packet has not be sent correctly, this leads to the retransmission of it.

(3) $$\delta =\delta_{Max}\times \left(1-BER\right)$$

**Figure 13:**
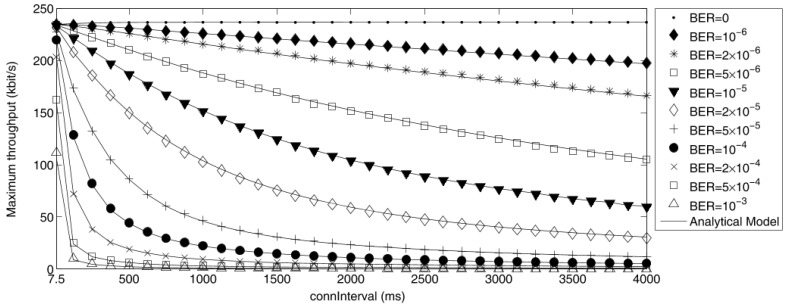
Maximum throughput of a Bluetooth Low Energy link for various *connInterval* (ranging between 7.5 ms and 4000 ms) and BER values (ranged from zero to 10${}^{-3}$): simulation (symbols) vs. analysis (lines). The simulation has been performed using 1,000,000 *connEvents* per each parameter set. Adapted from [65].

According to these considerations, Gomez et al. [65] estimated the maximum theoretical throughput of a Bluetooth Low Energy link obtained at different *connInterval* (ranging between 7.5 ms and 4000 ms) and for different levels of BER (ranged from zero to 10${}^{-3}$). The payload used is 20 bytes, which corresponds to the maximum acceptable by the BLE protocol, as described in Section 2.3. These results are shown in Figure 13. In absence of bit errors, the maximum BLE throughput estimated ($\delta_{Max}$) is 236.7 kbit/s, computed with Equation (2).

Nevertheless, the effective bitrate in common applications appears to be hardly lower than the estimated one, due to HW and firmware (FW) limitations. Indeed, especially in smartphones and tablets, the BLE chip is usually in the same Integrated Circuit (IC) of Wi-Fi and BR/EDR, and this implies a loss of performances if the three technologies are used simultaneously. Moreover, how the different vendors implement the BLE stack protocol in the FW influences the maximum number of packet per *connInterval* and also the minimum value that the *connInterval* can assume.

For example, most Android devices supports ~4 packets per *connInterval*, while most iOS devices supports up to six packets and a minimum *connInterval* of 20 ms [66], both depending on the specific device and operating system. Moreover, several producers of BLE controllers put a limitation on the maximum data packets sent per *connInterval*. For example, the BLE radio nRF51822 [67], developed by Nordic Semiconductor, can transmit up to six data packets per *connInterval*, making the maximum data rate limited to ~125 kbit/s [52]. In the SW simulator by STMicroelectronics [68], it is estimated how many data packets a BLE link, composed of BlueNRG-MS boards [69], can send per *connInterval*. This IC can send nine packets in a *connInterval* of 7.5 ms, which means it can reach a throughput of ~192 kbit/s; still, this value can be assumed as the upper bound of the bitrate, while the actual throughput is probably lower, due to other operations that the processor does in parallel.

To better understand the effective value of BLE throughput, it would be very useful to have more documents about the technical specification of the ICs provided by the different vendors. It is also important to know the characteristics relative to the different HW and Operation Systems (OS) for mobile and desktop devices. Moreover, it is important to know the real limits of throughput for several possible combination of *masters* and *slaves* with different HWs, OSs and FWs.

### 3.2. Piconet Size

Another important feature that can limit BLE performance (and wireless technologies in general) is the maximum number of devices, which can exchange data in the same star network, shown in Figure 12c.

Gomez et al. [50] investigated the maximum *piconet* size, which is the maximum number of *slaves* that a single *master* can handle. The BLE specification does not impose a specific limit on it, but it depends on the SW and HW characteristics, such as the amount of memory on the BLE IC, the antenna availability or some connection parameters (e.g., *connInterval*), the BER and the type of communication between *master* and *slaves*.

**Figure 14:**
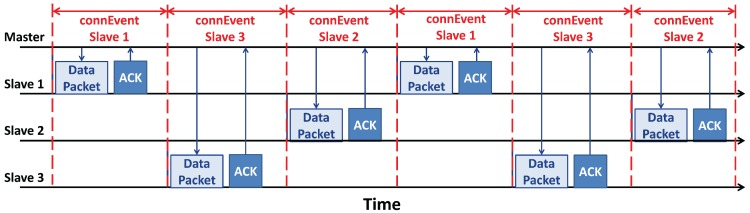
Multi *slave* communication in a network with a star topology (see Section 2.4). There is a *master* sending data with three *slaves*. This is the particular case of non-overlapping, i.e., when different *slaves* do not share the same interval of time for the communication with the *master*. In fact, as can be seen, all the *connEvents* are distributed without overlapping. The black arrows indicate how the time goes. The blue arrows outline the direction of the communication.

The *master* must reset its radio frequency every time it wants to reach a different *slave* and exchange messages with it [70]; for this reason, the *connInterval* parameter influences the maximum number of *slaves*. It is important to notice that different *slaves* belong to different *piconets* with the *master* in common; that means the connection parameters may also be different per each *slave*, and so per each *piconet*. Hence, the *master* needs the time to switch between the different *piconets* without an overlapping of their *connEvents*, as shown in Figure 14. It is also possible to have overlapping communications, but this will imply a loss of data during the transmission. For all the reasons explained, the number of *slaves* a *master* can support is inversely proportional to the maximum throughput of each connection; as a matter of fact, if a *master* needs to connect with a lot of *slaves*, it shall decrease the *connEvent* of each connection with a direct implication of less packets sent.

On the other hand, the maximum piconet size is independent of the *connSlaveLatency* parameter, because the inactive *connEvents*, due to *slave* latency and overlapping communications, cannot be used for connections with other *slaves* [50].

**Figure 15:**
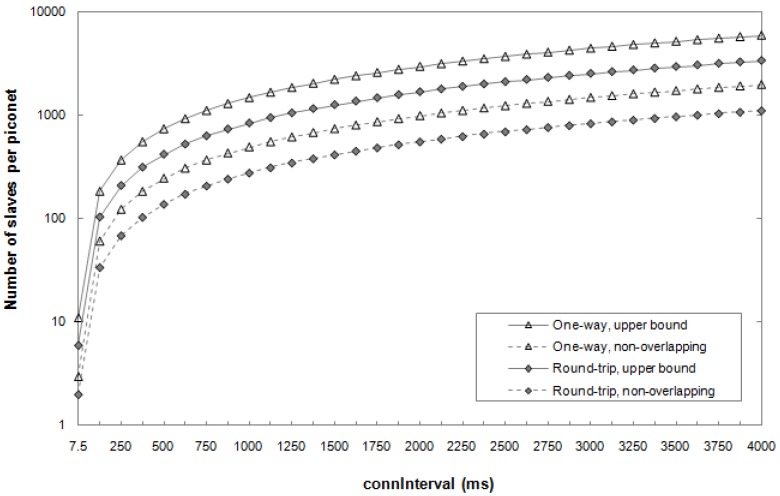
Theoretical maximum number of *slaves* per piconet for various types of interaction between devices, scheduling schemes and *connInterval*. The different types of interaction examined are the *one-way* and *round-trip* communications (described in Section 2.2.2). Moreover, the analysis has been done in the ideal case of non-overlapping communications, as well as in the case of overlapping, which denotes the upper bound limit. Adapted from [50].

The results they obtained are shown in Figure 15. They evaluated the maximum number of *slaves* in a *piconet* for the *one-way* and *round-trip* ATT interactions (Section 3.4), setting an upper bound obtained considering the ideal condition of a null BER. In addition, the case of non-overlapping has been evaluated, i.e., when two different *slaves* do not overlap their communication with the *master*, even when bit errors lead to retransmission.

They obtained that for a *connInterval* equal to 7.5 ms, the maximum number of *slaves* per *master* could be between two and 11, while, with a *connInterval* of 4000 ms, this number can theoretically be ~5900 in the *one-way* communication.

It would be useful to understand the real limit of the number of *slaves*, because this unique work estimates the theoretical results only with an analytical model. STMicroelectronics, for examples, affirms that their BLE devices can connect a maximum number of eight *slaves* [71]. Texas Instruments states the maximum number of *slaves* for their BLE IC, which is strictly dependent on the available RAM memory of the IC; in particular, this is due to the amount of heap memory allocated [72].

An important topic relative to BLE, and in general to wireless networks, is node synchronization. In fact sensor data fusion in a network is not possible if there is not a precise time synchronization between the different nodes of the network. The architecture of BLE provides non-deterministic delays, which make synchronization hard to implement. For this purpose, Rheinländer et al. [73] proposed two principles to obtain precise synchronization time stamps, based on the BLE IC power consumption.

### 3.3. Power Consumption

The main innovative characteristic of BLE, in comparison with Classic Bluetooth and other wireless technologies, is the low power consumption. Indeed, as described in Section 2.2, both in *broadcasting* and *connection* mode, the RF module turns on to send or receive data and then turns off in order to save energy consumption.

Bluetooth SIG affirms BLE has a current consumption, on average, lower than ANT+ by a factor of 1.5× and ZigBee of 2× [74].

STMicroelectronics provides a simulator [68] to evaluate and measure the power consumption during all possible states of the BLE IC RF module, BlueNRG-MS [69], also managing all the parameters.

To evaluate the power consumption of a BLE IC, it is necessary to evaluate the average current absorbed during the active phase in each modality of communication. In fact, on the other side, when the radio is in the sleep phase, the current consumption is approximately 1 μA (with 3 V of reference voltage level) [75].

In *broadcasting* mode, the *advertiser* sends a packet on the three advertising channels in each *advInterval*. Therefore, it is easy to notice that the consumption is directly proportional to the packet data length and to the number of channels used to communicate, while it is inversely proportional to the *advInterval*.

In scanning mode, the average current consumption is only related to the *Scan Window*, which determines the active phase, and the *scanInterval*, which indicates the time between two active phases.

Moreover, the energy consumption during the active scanning is higher than the passive scanning, described in Section 2.3, because the *advertiser* is set to listen to other requests from the *scanner*.

An analysis of the power consumption of a BLE *broadcasting* mode during the discovery process has been done by Liu et al. in [76]. They measured in detail the current consumption in each phase during the advertising mode using the BLE IC CC2541 [77] by Texas Instruments (using 3 V of reference voltage level). The results show that the current consumption in the active phase of advertising is ~10.9 mA. That means that the total current consumption during advertising, with the minimum *advInterval* of 20 ms, is ~2.3 mA. Moreover, they validated an analytical model of energy consumption in advertising mode, depending on the variable parameters that define this type of communication (*advInterval*, *scanInterval* and *Scan Window*).

**Figure 16:**
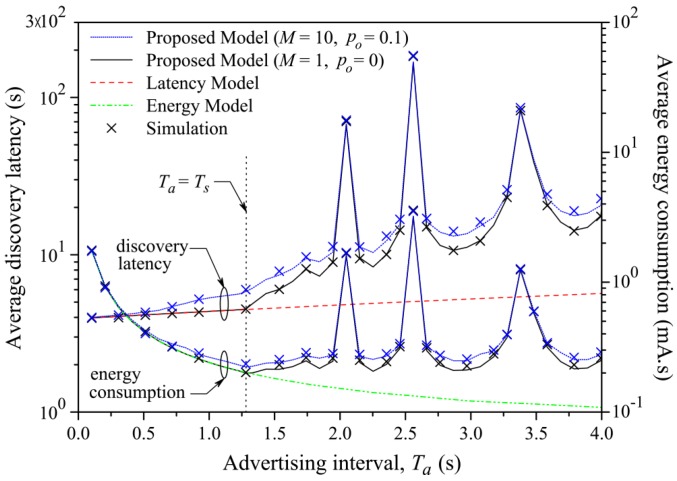
Average discovery latency and energy consumption of the *advertiser* according to $T_{a}$ ($T_{s}$ = 1.28 s and *T* = 10.24 s) [78]. The green line shows the model proposed by [76], while the black and blue lines positioned in the lower part show the models analyzed in [78]. The crosses represent the simulation based on the theoretical model. As can be noticed, when $T_{a}$ > $T_{s}$, the two results do not agree anymore. On the other side, the red line [79] and the two blue and black lines positioned in the upper part of the figure represent the data relative to the latency, analyzed in Section 3.4. *M* is the number of pairs of *scanners* and *advertisers* existing in the communications, while $p_{0}$ is the failure probability due to the interference with other devices. Adapted from [78].

**Figure 17:**
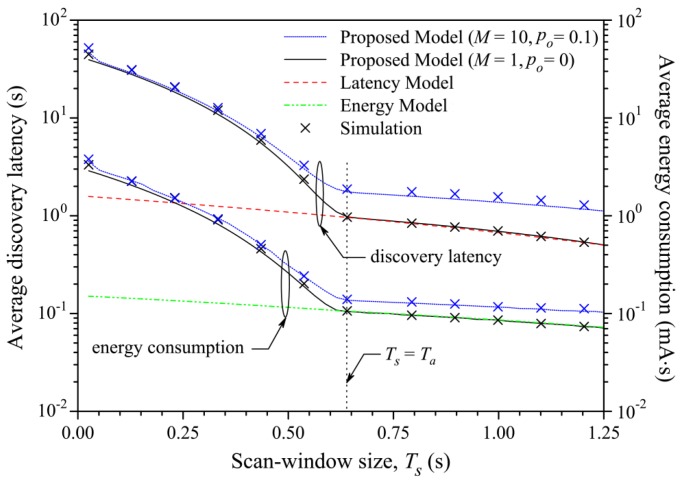
Average discovery latency and energy consumption of the *advertiser* according to $T_{s}$ ($T_{a}$ = 0.64 s and *T* = 2.56 s) [78]. The green line shows the model proposed by [76], while the black and blue lines positioned in the lower part show the models analyzed in [78]. The crosses represent the simulation based on the theoretical model. As can be noticed, when $T_{s}$ < $T_{a}$, the two results do not agree anymore. On the other side, the red line [79] and the two blue and black lines positioned in the upper part of the figure represent the data relative to the latency, analyzed in Section 3.4. *M* is the number of pairs of *scanners* and *advertisers* that can communicate with each other, while $p_{0}$ is the failure probability due to the interference with other devices. Adapted from [78].

A comparison and a new model to evaluate power consumption during the discovery process has been proposed by [78]. They found that the results about the energy consumption are comparable with [76], but they proposed a more precise model when the *advInterval* is higher than the *Scan Window*; see Figure 16 and Figure 17. They assumed that the operating voltage of a device is constant, so that the energy consumption can be assessed as the device current multiplied by the elapsed time.

For a large number of devices in a discovery process, there is the possibility of collision and hence of an unsuccessful discovery. For this reason, Kim et al. [80] proposed an enhanced discovery mechanism by employing a backoff strategy for BLE devices, in order to reduce collisions during advertising processes. The results they have shown in this paper highlighted that the proposed mechanism improves the performance in terms of energy consumption, as well as latency (Section 3.4).

A power analysis and a battery life estimation in *advertising* mode for a concrete application has been done by Fafoutis et al. in [3], using the nRF51822 BLE IC. They extrapolate the power profile and the energy consumption when transmitting a triple BLE advertisement, which goes from around 30 μJ to less than 40 μJ, depending on the transmission power (range from −21 dBm to 5 dBm).

During the connection mode, it is more difficult to examine the power consumption because it is strongly variable, depending on several parameters, such as the packet payload, the *connInterval*, the number of *slaves* per *master*, the type of communication (*one-way* or *round-trip*), and so on.

In [68,70,81,82], the wave form of the current in the BLE IC is modeled in detail and described in order to calculate the value of the average current in the *connEvent*. The average current consumption, estimated for the minimum *connInterval* of 7.5 ms, is ~3200 μA, while the consumption with a *connInterval* of 2 s is about ~13 μA. The operating voltage for the BLE IC is 3 V.

**Figure 18:**
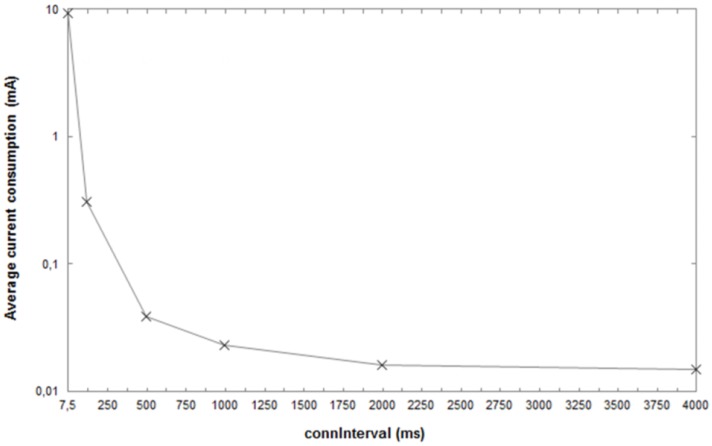
Average current consumption, per variation of *connInterval*, measured in a CC2540 *slave*. The operating voltage of this IC is 3 V. It is a *one-way* communication with *connSlaveLatenxy* = 0. The Texas Instruments BLE module CC2540 [83] has a USB interface that implies a more relevant drawn current compared to I2C or SPI interfaces (e.g., the CC2541 [77] module has an I2C interface). A BLE module with a USB interface could be a good reference for a *central* node rather than a *peripheral* one. Adapted from [50].

In addition to this, the relation between the energy consumption and the *ConnInterval* has been evaluated in [50,70]. It has been seen that the average consumptive current decreases exponentially at the raising of the *connInterval*, supposing a BER = 0, as shown in Figure 18. However, bit errors may significantly affect the current consumption, depending on each *connInterval* and *connSlaveLatency*.

Feng et al. [84] have also analyzed the variability in power consumption depending on the effective number of byte per packet, comparing two different IC: one developed by Texas Instruments, the CC2541 [77], and one by Nordic Semiconductor, the nRF51822 [67]. These two BLE IC use different types of architecture: the first is based on Intel 8051, while the second is an ARM MCU; it would be also interesting to investigate how performance varies in these different microcontroller architectures.

According to the results exposed above, Gomez et al. [50] estimated the life-time of a BLE device powered by a coin cell battery; it ranges between 2.0 days and 14.4 years, depending on the specific application.

**Figure 19:**
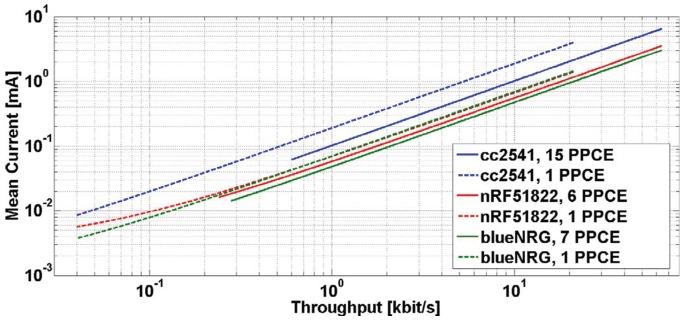
Current consumption as a function of throughput for different BLE ICs. This value is computed using the maximum and the minimum number of packets per *connEvent* (PPCE) each device may support. Adapted from [85].

An interesting study that compares the power consumption of different BLE IC existing on the market has been done in [85]. As can be seen in Figure 19, they measured the mean current consumption as a function of throughput; moreover, each BLE IC has been evaluated using the maximum and the minimum number of packets per *connEvent* it may support. The modules used are BlueNRG [86] by STMicroelectronics, cc2541 [77] by Texas Instruments and nRF51822 [67] by Nordic Semiconductor.

Furthermore, Aguilar et al. [87] modeled the BLE communication, both in *advertising* and *connection* mode, in relation with several communication parameters. An interesting parameter they use to analyze energy consumption of BLE is the energy cost, i.e., how much energy per bit (in terms of Joules per collected bit) BLE uses.

These different works agree with the same results, so it is possible to affirm that the current consumptions, theoretically estimated and experimentally validated, during packet transmission in *advertising* and *connections*, are almost equal. The only variable factors are the specific BLE IC and the FW vendor implementation; these two factors can slightly influence the power consumption of the device.

### 3.4. Latency

In the context of communication, the latency is the interval of time a data packet needs to be transmitted from a node to another one, and vice versa.

In some studies, the latency has been modeled and evaluated during the discovery phase, where a node scans other nodes to connect with and also during a connection [79,88,89,90]. The discovery latency refers to the interval for the *advertiser* to enter into advertising mode up to the point when the advertising information is received by an *initiator*. The discovery latency as function of the *AdvInterval* and the *SccanInterval*, described in Section 2.2.1, has been modeled and evaluated in [79]. The authors defined two new parameters:

(4) $$\alpha =\frac{AdvInterval}{T}$$

(5) $$\beta =\frac{ScanInterval}{T}$$

**Figure 20:**
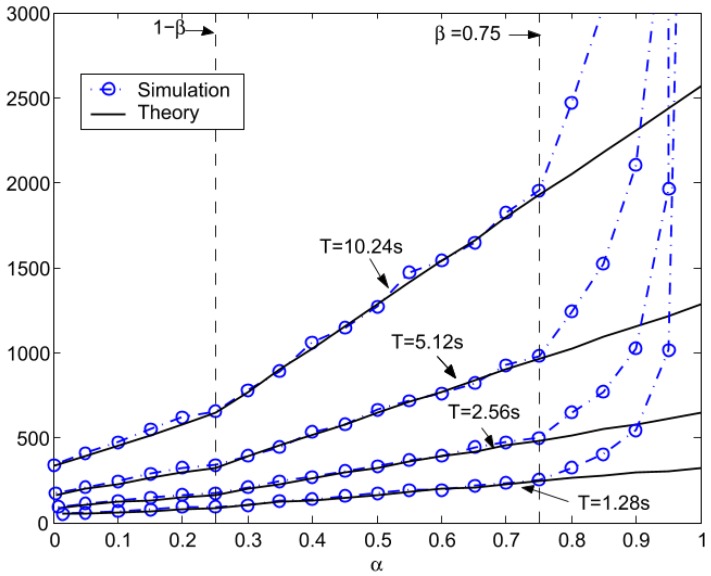
Average latency comparison with varied advertising duty ratio (α) and a fixed scanning duty ratio (β = 0.75). Adapted from [79].

**Figure 21:**
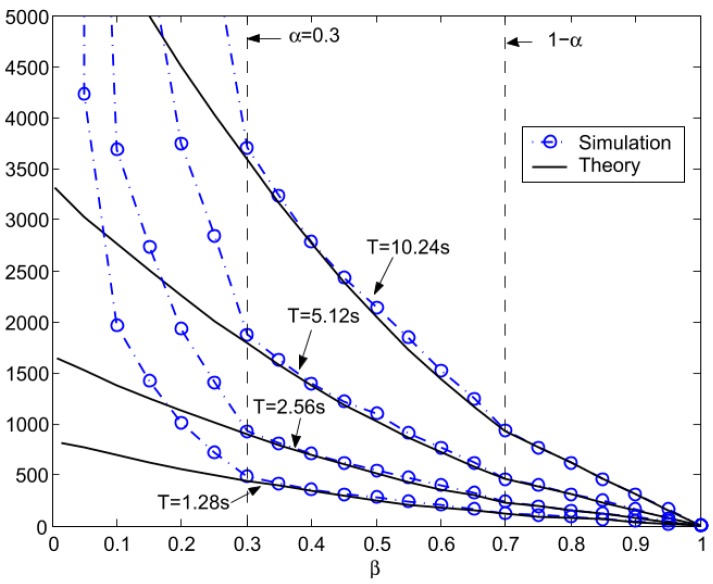
Average latency comparison with varied scanning duty ratio (β) and a fixed advertising duty ratio (α = 0.3). Adapted from [79].

They obtained the average latency, both theoretical and simulated, for fixed values of α and variations of β and vice versa, as shown in Figure 20 and Figure 21.

**Figure 22:**
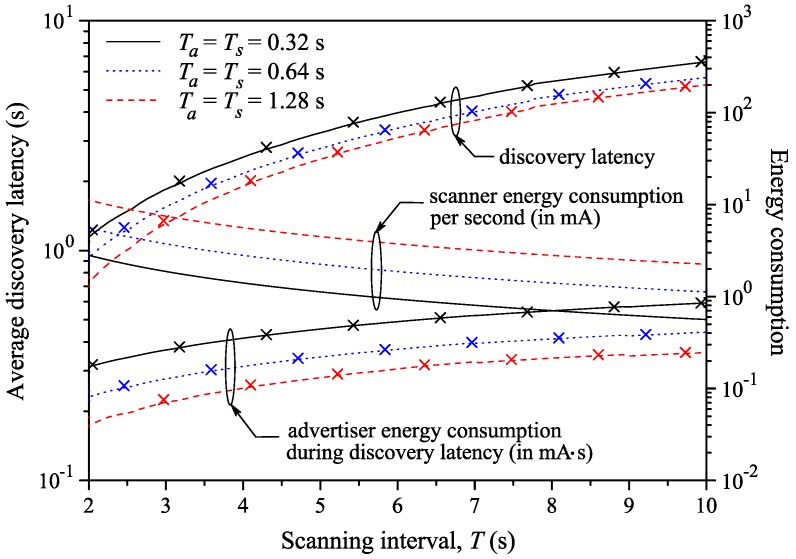
Average discovery latency and energy consumption of the *advertiser* according to $T_{a}$ ($T_{s}$ = 1.28 s and *T* = 10.24 s) [78]. The green line shows the model proposed by [76], while the black and blue lines positioned in the lower part show the models analyzed in [78]. The crosses represent the simulation based on the theoretical model. As can be noticed, when $T_{a}$ > $T_{s}$, the two results do not agree. On the other side, the red line [79] and the two blue and black lines positioned in the upper part of the figure represent the data relative to the latency, analyzed in Section 3.4. Adapted from [78].

**Figure 23:**
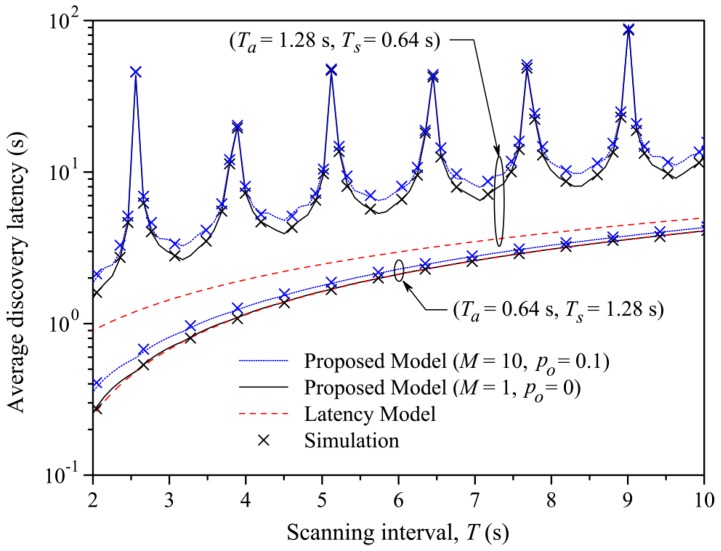
Average discovery latency of the *advertiser* according to different values of *Scan Interval*. Blue and black curves indicate the theoretical model and the simulation proposed in [78], while the red lines represent the latency model of [79]. *M* is the number of pairs of *scanners* and *advertisers*, which can communicate with each other, while $p_{0}$ is the failure probability due to the interference with other devices. Adapted from [78].

A similar study about the discovery latency has been done in [78]. They also proposed a general model for analyzing BLE in the discovery process. This model is also validated using a simulation compared with the experimental results obtained by [79]. They found that the results of the two works are comparable, noticing that the average discovery latency, varying *advInterval*, *scanInterval* and *Scan Window*, goes from 1 to 30 s. They also highlighted that [79] fails to model the discovering process when *advInterval* is higher than *Scan Interval*. These results are shown in Figure 16, Figure 17, Figure 22 and Figure 23.

In order to decrease collisions and unsuccessful discoveries, Kim et al. [80] proposed a new discovery mechanism, already mentioned in Section 3.3, which improves BLE performance in terms of latency.

**Figure 24:**
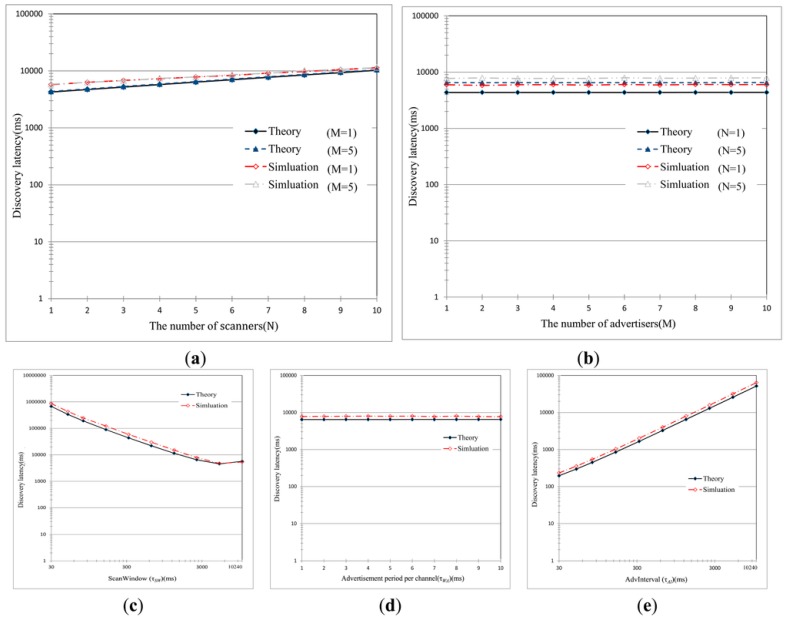
Mean discovery latency with various parameter settings. (**a**) The mean discovery latency as the number of *scanners* is increased ($\tau_{SI}$ = 10,240, $\tau_{SW}$ = 2560, $\tau_{WA}$ = 10, $\tau_{AI}$ = 1280); (**b**) the mean discovery latency as the number of *advertisers* increases ($\tau_{SI}$ = 10,240, $\tau_{SW}$ = 2560, $\tau_{WA}$ = 10, $\tau_{AI}$ = 1280); (**c**) the mean discovery latency as *ScanWindow* ($\tau_{SW}$) is varied ($\tau_{SI}$ = 10,240, $\tau_{WA}$ = 10, $\tau_{AI}$ = 1280, M = 5, N = 5); (**d**) the mean discovery latency as $\tau_{WA}$ is varied ($\tau_{SI}$ = 10,240, $\tau_{SW}$ = 2560, $\tau_{AI}$ = 1280, M = 5, N = 5); (**e**) the mean discovery latency as *AdvInterval* ($\tau_{AI}$) is varied ($\tau_{SI}$ = 10,240, $\tau_{SW}$ = 2560, $\tau_{WA}$ = 10, M = 5, N = 5). $\tau_{SI}$ is the *scanInterval*; $\tau_{SW}$ is the *ScanWindow*; $\tau_{AI}$ is the *advInterval*; M is the number of *advertiser*; N is the number of *scanners*. Adapted from [88].

Furthermore, Cho et al., in [88,89], studied in theory and in simulation how the discovery latency varies in relation to some network parameters (shown in Figure 24), in addition to the parameters observed above. For example, they evaluate the latency in comparison with the number of *scanners* and *advertisers*. Their results are shown in Figure 24. Similar analyses have also been done by Contrera et al. in [91], in which they evaluate the discovery latency changing the *advertising* and *scanning* parameters.

In *connections*, latency can be measured either as the *one-way* delay time, that is the time a packet spends from the source to the destination, or as the *round-trip* delay time, i.e., the sum of the *one-way* latency from the source to the destination and the *one-way* latency in the opposite way for the response.

**Figure 25:**
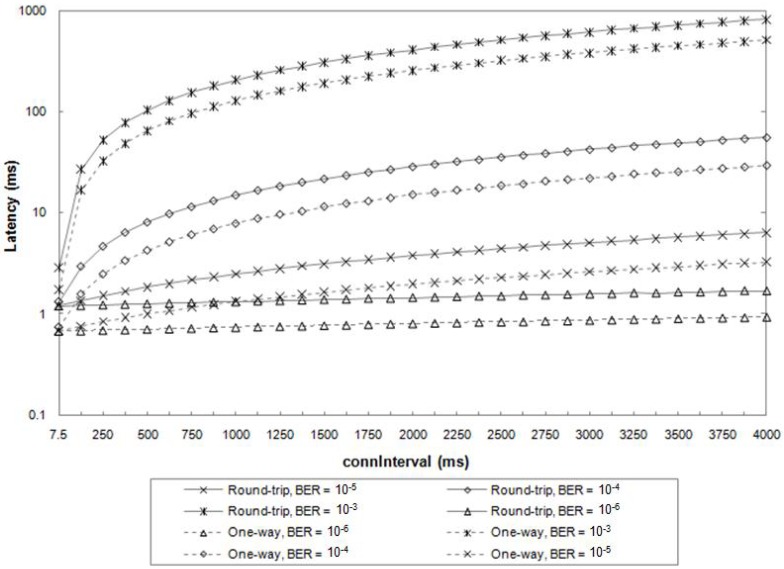
Average latency for *one-way* and *round-trip* message exchanges, for various *connInterval* and BER values. Adapted from [50].

Gomez et al. [50] also simulated the latency during connections between a *master* and a *slave*, in one-way and round-trip ATT communications, in relation to *connInterval* and BER values, as shown in Figure 25.

The figure shows how the latency, with a reasonable BER (included between $10^{-6}$ and $10^{-4}$), is included in a range of values from 0.6 ms to 10 ms, both in *one-way* and *round-trip* communications.

In [92], the authors developed a new connection establishment method for Mobile Ad Hoc Networks (MANET). These networks usually use a method to establish the connection based on Classic Bluetooth. On the other side, this paper shows that the new modality proposed, firstly based on BLE and then switching on Classic Bluetooth, is almost 75% shorter with an energy consumption 18% lower.

### 3.5. Range

The maximum range a BLE RF module may reach is strictly linked to type of antenna used, e.g., PCB, chip or whip [93,94] and to the *path loss*, i.e., a measure of how much the power of the radio signal is reduced along the path starting from the transmitter and ending in the receiver. The *path loss* is defined as the difference between the radio power of the transmitter, described in Section 2.1.1, and the receiver sensitivity, both measured in dBm [31,55].

**Figure 26:**
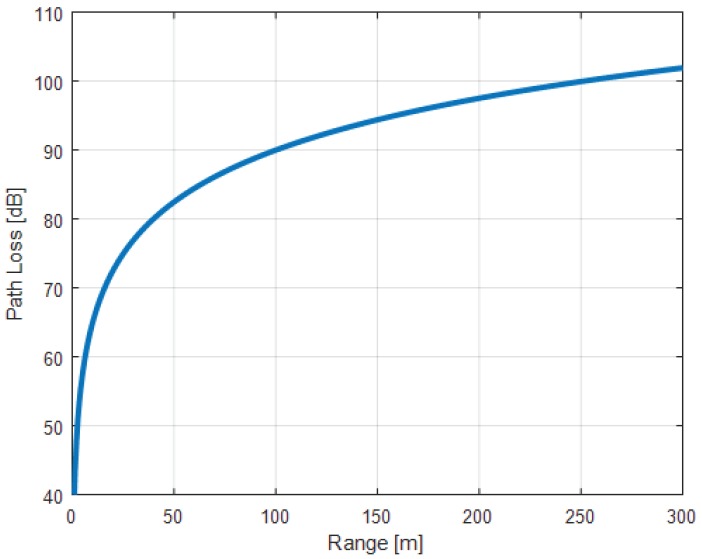
A graphic representation of *path loss* , obtained from Equation (6). Adapted from [55].

The correlation between the *path loss* and the distance (*d*) is described in [55] with Equation (6) and shown in Figure 26. This equation is valid only for an isotropic antenna and ignores any losses, reflection, noise or obstacles existing in the environment [55].

(6) pathloss=40+25×log(d)

According to the BLE specification [53], a BLE receiver must have a sensitivity lower than or equal to −70 dBm, while a transmitter could have its radio power included in a range from −20 dBm (0.01 mW) to +20 dBm (100 mW).

Concretely, an STMicroelectronics BLE IC could set its radio power between −18 dBm and +8 dBm [69]. For example, with a transmitter using a power of +8 dBm and a receiver having a sensitivity of −70 dBm, the *path loss* is +78 dB. With this value of *path loss*, this BLE communication can theoretically reach a distance of ~33 m.

Relative to the estimation of the maximum distance reachable by a BLE link, there is not a practical evaluation in the literature that analyzes the effective range of a BLE RF module.

BLE is also used to detect the distance between different nodes of the network. In fact, RF modules in general provide a feature called Received Signal Strength Indicator (RSSI) that indicates the power strength of the Tx signal received by the Rx node. Several studies indicate empirically the relationship between RSSI and distance ([23,29,30,33,34]), but [22] finds a model that can be used to calculate the distance directly from the RSSI, as described in the following equation:

(7) RSSI=−10×N×log(d)+a

where *N* is a constant assumed as one, *d* is the distance in meters between the two devices and *a* is the power of the Tx at a one-meter distance.

In [31,32,91], a more precise method is described to compute the distance using the RSSI value. In particular, [91] also analyses and evaluates the operation and performance of BLE, changing the main parameters of *advertising* and *scanning*. They also show how the cut-off frequency of the signal filtering influences the uncertainty in the position estimation.

## 4. Discussion

The aim of this review is to describe the BLE stack and the functioning of the protocol of this wireless communication technology. Then, we show the data already existing in the literature, highlighting the agreements and the contrasts of the results and proposing the main topics to study in depth, in order to obtain complete knowledge about them. Finally, from the literature analysis, it was possible to find the essential parameters helpful to understand how to set BLE depending on the specific application.

As concerns power consumption (Section 3.3), the literature offers a wide number of articles that give results during the different typologies of BLE communication. These results generally agree on the amount of energy BLE consumes, and they define how it varies, changing the different protocols’ settings. One important aspect that is currently missing in the literature is to accurately measure consumptions during all the different operations. In addition to this, it is important to notice that energy consumption is easy to model starting from the known waveform of a BLE communication.

Regarding latency (Section 3.4), there are many studies that agree in their results. These papers model this parameter in all of the several typologies of communication and varying all the protocol settings. Therefore, it could be said that the information, obtained from the literature, is almost exhaustive.

On the other hand, the maximum data throughput (Section 3.1), the maximum number of *slaves* per *piconet* (Section 3.2) and the maximum range of transmission (Section 3.5) are not deeply investigated. These are important parameters that permit understanding how to push BLE to its limits, opening new horizons in other possible application fields. The results existing in the literature are not exhaustive and do not agree in the values they propose, also because they are strictly dependent on the HW and FW of the specific BLE IC, being different from vendor to vendor.

Some of the results existing in the state of the art are theoretical, and they fix upper-bounds that are not pragmatic or realistic, because the HW, the SW and the FW put some limits that hardly underrate the performance of the communication.

An important factor is that the information provided by the vendors about their IC is often not exhaustive in order to know the effective performance of the BLE chip implemented in the HW systems. These important pieces of information are for example the maximum number of packets per *connInterval*, the maximum number of *slaves* and the knowledge of the settable connection parameters, such as the minimum *connInterval*. These limits are imposed by the HW and the FW, but also by the Application libraries implemented in the OS. It would be interesting to analyze the different devices available on the market, produced by several vendors; e.g., in particular, how microcontrollers of different families (ARM- or 8051-based MCUs) influence the BLE communication.

On the other hand, the existing applications in the state of the art in which BLE is implemented do not push it to its limit, so it is not easy to know what its effective performance is.

With this work, we want to provide a comprehensive review of BLE performance, by stressing what is known and what has to be further investigated, in order to propose a roadmap for systematic experimental validation of its main characteristics and pave the way for further studies, which can describe the actual limits of BLE technologies.

As already said before, what we know about power consumption is mainly obtained from theoretical models, starting from the known BLE waveform. These models are accurate, but they still need a concrete and systematic evaluation of the real energy consumption of the BLE IC, during the different operations (e.g., *broadcasting, listening, connections*), varying the protocol settings (e.g., *connInterval, scanInterval, advInterval*), throughput and the radio power. Moreover, it would be very interesting to investigate how the power consumption depends on the different vendor BLE ICs existing on the market, since it could be slightly dependent on the specific HW and FW.

Of particular interest will be the experimental validation of throughput in correlation with the number of *slaves*. As a matter of fact, it is interesting to understand what is the limit in terms of bitrate and how it changes in relation with a variable number of nodes, with different connection parameters, such as *connInterval*, but also using different network topologies. It will be useful also to measure these data, varying the distance between nodes and the radio power, in order to verify the existing models. Finally, a detailed analysis of power consumption in relation to the variations of all these parameters could help to identify the optimal configuration of BLE networks for different applications.

Another important topic relative to BLE, which needs deep investigations, is node synchronization. As a matter of fact, as already said in Section 3.2, many applications that involve sensor networks need a precise time synchronization between the different nodes. The architecture of BLE provides non-deterministic delays, which make synchronization hard to implement.

These analyses will help to understand what is the effective pool of possible applications in which BLE could work, exalting its capacities and behaviours between the several wireless communication technologies. 

## Figures and Tables

**Table 1:** This table resumes all the parameters introduced in Section 2, which are responsible for the BLE performance analyzed in Section 3. The first column contains the name of the parameter and the subsection where it is defined; the second describes this parameter; the third contains the performances influenced by this specific parameter and the subsection where they are described in detail.

Key-Parameters	Description	Characteristic Addressed
*connEvent* Section 2.2.2	The time in which two devices exchange packets	Throughput Section 3.1, Latency Section 3.4, Power consumption Section 3.3, Piconet size Section 3.2
*connInterval* Section 2.2.2	The time between two consecutive *connEvents*	Throughput Section 3.1, Latency Section 3.4, Power consumption Section 3.3, Piconet size Section 3.2
*Radio Idle* Section 2.2.2	The period between two consecutive *connEvents*, when the communication is off	Throughput Section 3.1, Latency Section 3.4, Power consumption Section 3.3, Piconet size Section 3.2
*connSlaveLatency* Section 2.2.2	Amount of *connEvents* which can be skipped avoiding the risk of disconnections	Piconet size Section 3.2, Latency Section 3.4
*advInterval* Section 2.2.1	The rate at which advertising packets are sent	Power consumption Section 3.3, Latency Section 3.4
*scanInterval* Section 2.2.1	The rate at which the scanner’s radio turns on	Power consumption Section 3.3, Latency Section 3.4
*scanWindow* Section 2.2.1	The amount of time the radio keeps on scanning	Latency Section 3.4
*round-trip* Section 2.2.2	The time to send a data packet and an ACK.	Throughput Section 3.1, Latency Section 3.4
*one-way* Section 2.2.2	The time to send a packet in *notification* mode	Throughput Section 3.1, Latency Section 3.4
*payload* Section 2.3	Number of bytes per each data packet, that is 20 bytes	Throughput Section 3.1
*piconet* Section 2.4	A basic BLE network, composed by a *master* and a *slave*	Piconet size Section 3.2
*scatternet* Section 2.4	A network where a device performs *master* and *slave* simultaneously.	Piconet size Section 3.2

## Abbreviations

The following abbreviations are used in this manuscript:

6LoWPAN	IPv6 over Low power Wireless Personal Area Networks
AA	Access Address
ACK	Acknowledgment
AdvA	*Advertiser*’s device Address
AdvData	Advertising Data
*AdvInterval*	Advertising Interval
AES	Advanced Encryption Standard
App	Application Layer
ATT	Attribute Protocol
BER	Bit Error Rate
BLE	Bluetooth Low Energy
BR/EDR	Bluetooth Basic Rate/Enhanced Data Rate or Classic Bluetooth
ChSel	Channel Selection
ConnData	Connection Data
*ConnEvent*	Connection Event
*ConnInterval*	Connection Interval
*ConnSlaveLatency*	Connection Slave Latency
*ConnSupervisionTimeout*	Connection Supervision Timeout
CRC	Cyclic Redundancy Check
CPU	Central Process Unit
FW	Firmware
GAP	Generic Access Profile
GATT	Generic Attribute Profile
HCI	Host Control Interface
HW	Hardware
IC	Integrated Circuit
IETF	Internet Engineering Task Force
IoT	Internet of Things
IPv6	Internet Protocol version 6
ISM	Industrial Scientific and Medical
L2CAP	Logical Link Control and Adaptation Protocol
LE	Low Energy
LE 1M PHY	Low Energy 1 Mbps Physical Layer data rate
LE 2M PHY	Low Energy 2 Mbps Physical Layer data rate
LL	Link Layer
LLID	Logical Link Identifier
LM	Link Manager
MANET	Mobile Ad hoc Networks
MD	More Data
MIC	Maximum Transmission Unit
M-IMU	Magneto-Inertial Measurement Unit
MTU	Maximum Transmission Unit
NESN	Next Expected Sequence Number
OS	Operative Systems
PDU	Protocol Data Unit
PHY	Physical Layer
PPCE	Packets Per Connection Event
PRE	Preamble
RFCOMM	Radio Frequency Communication
RFU	Reserved for Future Use
RSSI	Received Signal Strength Indicator
RxAdd	Receiver Address
*ScanInterval*	Scanning interval
SDP	Service Discovery Protocol
SIG	Special Interest Group
SMP	Security Manager Protocol
SN	Sequence Number
SW	Software
TxAdd	Transmitter Address
UUID	Universal Unique Identifier
